# Metabolic Fate and Distribution of 2´‐Fucosyllactose: Direct Influence on Gut Microbial Activity but not on Brain

**DOI:** 10.1002/mnfr.201900035

**Published:** 2019-06-07

**Authors:** Sabine Kuntz, Clemens Kunz, Christian Borsch, Enrique Vazquez, Rachael Buck, Martina Reutzel, Gunter Peter Eckert, Silvia Rudloff

**Affiliations:** ^1^ Institute of Nutritional Sciences Justus‐Liebig University Giessen 35392 Giessen Germany; ^2^ Institute of Pharmacology Goethe‐University Frankfurt 60438 Frankfurt am Main Germany; ^3^ Discovery R&D, Abbott Nutrition 18004 Granada Spain; ^4^ Discovery R&D, Abbott Nutrition Columbus OH 43219 USA; ^5^ Department of Pediatrics Justus‐Liebig University Giessen 35392 Giessen Germany

**Keywords:** ^13^C‐labelled 2´fucosyllactose, brain enrichment, germ‐free mice, metabolism, microbiota

## Abstract

**Scope:**

2´‐Fucosyllactose (2´FL) is an abundant oligosaccharide in human milk. It is hypothesized that its brain enrichment is associated with improved learning. Accumulation of 2´FL in organs, biological fluids, and feces is assessed in wild‐type and germ‐free mice.

**Methods and results:**

^13^C‐labelled 2´FL is applied to NMRI wild‐type mice intravenously (0.2 g kg^−1^) or orally (1 g kg^−1^), while controls receive saline. Biological samples are collected (0.5–15 h) and ^13^C‐enrichment is measured by elemental analysis isotope ratio mass spectrometry (EA‐IRMS). After oral application, 2´FL is primarily eliminated in the feces. ^13^C‐enrichment in organs including the brain follows the same pattern as in plasma with a maximum peak after 5 h. However, ^13^C‐enrichment is only detected when the ^13^C‐2´FL bolus reaches the colon. In contrast, in germ‐free mice, the ^13^C‐bolus remains in the intestinal content and is expelled via the feces. Furthermore, intravenously applied ^13^C‐2´FL is eliminated via urine; no ^13^C‐enrichment of organs is observed, suggesting that intact 2´FL is not retained.

**Conclusions:**

^13^C‐enrichment in brain and other organs after oral application of ^13^C‐2´FL in wild‐type mice indicates cleaved fucose or other gut microbial 2´FL metabolites may be incorporated, as opposed to intact 2´FL.

## 1 Introduction

Human milk oligosaccharides (HMO) belong to the major components in human milk and are characterized by their large variety and high amounts during the first months of lactation.^1^, ^2^, ^3^, ^4^ Influencing factors are the mother's genetic background and the stage of lactation.^1^, ^2^, ^4^, ^5^, ^6^ It is estimated that there are about 150–200 different structures, among which 2´fucosyllactose (2´FL) is the most abundant component in human milk from women with a positive secretor status (about 70–80% in Europeans), i.e., those expressing fucosyltransferase 2.^2^, ^5^, ^6^, ^7^, ^8^

HMO exert a variety of functions which, so far, have primarily been shown in vitro and in animal studies, although just recently the first human studies with HMO added to infant formula have been reported.^9^, ^10^, ^11^, ^12^

One of the most intensely investigated functions is their bifidogenic effect^13^, ^14^, ^15^ and their protecting influence against infections.^4^, ^16^, ^17^ Also, 2´FL acts as an anti‐inflammatory and immune‐modulating HMO.^18^, ^19^, ^20^ Clinical research showed that 2´FL reduces systemic inflammatory cytokines to levels more like breast fed infants, and attenuates peripheral blood mononuclear cell‐derived inflammatory cytokines after ex vivo stimulation with respiratory syncytial virus.^21^

Moreover, it has been found that human milk feeding is associated with improved cognitive development.^22^ Within this context, HMO are discussed as one of the responsible factors. It is hypothesized that acidic oligosaccharides in human milk would increase sialic acid content in brain gangliosides with succeeding effects on brain functions.^23^, ^24^ Furthermore, changes in hippocampal long term potentiation (LTP), a synaptic model of memory which is used as a marker for signal transmission between neurons,^25^ was influenced by fucose‐containing macromolecules in vivo^26^ and by 2´FL in vitro.^27^ Similar to these observations, it has recently been demonstrated that LTP was enhanced in rats receiving oral 2´FL but not after fucose application.^28^ Therefore, the gut–brain axis, especially the vagus nerve, is considered to be a key component in this crosstalk. However, there is limited information available on the metabolism and physiologic function of 2´FL concerning the brain, particularly with regard to its effects on memory and learning. Currently, it is unknown whether 2´FL itself or a metabolite is responsible for the observed effects on LTP. To achieve such an effect, 2´FL or its metabolites may need to accumulate in the corresponding brain regions. This, however, has not been investigated so far.

From metabolic studies in infants, it is known that 2´FL in milk from secretor mothers is excreted via the infant´s urine.^29^, ^30^ In addition, using stable isotopes, in vivo ^13^C‐labelling of HMO via a ^13^C‐galactose bolus to breastfeeding mothers has shown that HMO, including 2´FL, can be detected in urine of their infants.^31^, ^32^, ^33^, ^34^ These results imply that 2´FL was absorbed, passed the liver and reached the peripheral circulation before being excreted via the kidney. Indeed, small amounts of 2´FL were detected in plasma of breastfed infants of secretor mothers but not in infants of non‐secretor mothers or in formula‐fed infants.^21^ Notwithstanding, the majority of ingested HMO are considered to reach the colon where they may be used as substrates for intestinal bacteria, for example, for some strains of *Bifidobacteria*.^35^, ^36^, ^37^, ^38^, ^39^ It has been shown for *Bifidobacterium longum subsp. infantis* that released fucose can be further metabolized to ATP via the fructose‐6‐phosphate phosphoketolase pathway, with acetate and lactate as end products. In contrast, extracellular glycosyl hydrolases in *Bifidobacterium bifidum* generate metabolites that may serve as substrates for *B. infantis*. Hence, co‐existing or cross‐feeding effects influence HMO metabolism.^40^, ^41^ In infants, the development of the gut microbiota during the first month of life showed that colonization of FL‐utilizing *Bifidobacteria* is associated with an altered metabolite profile and changes in microbiota composition.^14^ Infants of non‐secretor mothers showed a lower abundance of *Bifidobacteria* and more *Enterobacteriaceae* with concomitant lower acetate concentrations.^14^ Such changes in microbiota interactions through specific metabolites have been suggested to exert a variety of beneficial effects on the host, and possibly on the gut–brain axis as well.

The overall aim of our study was to investigate whether ^13^C‐labelled 2´FL is absorbed or metabolized in the intestine and subsequently enriched in the brain in a wild‐type mouse model. Germ‐free mice were used to evaluate microbiota‐related effects.

## 2 Experimental Section

### 2.1 Materials

Isotopically labelled 2´FL containing one ^13^C in the fucose‐ring ([1‐^13^C_1_]‐2´FL [^13^C‐2´FL]) was obtained from Elicityl (Crolles, France).

### 2.2 Dosage Information

The dose of 2´FL used in the animal models corresponds to physiological conditions in humans. A newborn infant may receive 2 g 2´FL per liter with mature milk; the average volume of milk an infant receives a day is estimated to be 850 mL.^42^ Hence, a 4 kg infant would receive about 500 mg 2´FL per kg body weight.

### 2.3 Animal Models

#### 2.3.1 Intravenous Application of ^13^C‐Labelled 2´FL to Wild‐Type NMRI Mice

Male NMRI mice (8 weeks old, 37–43 g body weight) were purchased from Charles River Laboratories (Sulzfeld, Germany). Mice were housed in groups of five animals with free access to water and food (Altromin Spezialfutter GmbH & Co KG, Lage, Germany). On experimental days animals were weighed and each animal received ^13^C‐2´FL (treated, *n* = 5) or 0.9% NaCl (controls, *n* = 3) three times every 6 h through the tail vein, that is, in total 200 mg ^13^C‐2´FL per kg body weight. From the time of injection, animals were kept individually in metabolic cages (Tecniplast Laboratory Animal Equipment, Hohenpeissenberg, Germany). 24 h after the first injection, animals were anesthetized with isoflurane (Forene 100% v/v, Abbvie, Ludwigshafen, Germany) and pentobarbital (Narcoren, Halbergmoos, Germany) by intraperitoneal injection. A blood sample of each animal was taken from the retrobulbar plexus and was centrifuged at 1000 × *g* at 4 °C for 10 min to obtain plasma. To avoid plasma contamination of organs, the body was perfused with saline. Prior to the perfusion process, withdrawal effects were checked with forceps and the chest was opened with a midline skin incision. The abdomen was carefully opened and a small needle placed into the left ventricle. Perfusion was completed when the color of the perfusate turned from red to colorless. The brain was quickly removed and placed on ice followed by separating stem, cerebellum, and cerebrum. Other organs (liver, heart, spleen, and kidney) were also removed. All samples were snap‐frozen in liquid nitrogen and kept at −80 °C until analysis. Furthermore, urine and feces left in the metabolic cages were collected and kept at −80 °C until analysis.

#### 2.3.2 Oral Application of ^13^C‐Labelled 2´FL to Wild‐Type NMRI Mice

Male NMRI mice (8 weeks of age, 36–47 g body weight) were housed as described above. On the day of experiments animals (*n* = 40) received either a single dose of 1 g ^13^C‐2´FL per kg body weight (treated, *n* = 5 per time point) or saline as the vehicle (controls, *n* = 3 per time point) via oral gavage. Compared to the intravenous application, the gavage dose was increased due to an expected low absorption rate of 2´FL in the gastrointestinal tract. Animals were kept individually in metabolic cages and sacrificed after 0.5, 1, 2, 3, 5, 9, and 15 h. Perfusion and sample collection was conducted as described above. Furthermore, the small intestine (SI) was cut into three pieces of equal length; the large intestine (LI) was taken separately. Intestinal content was collected from each segment. Furthermore, urine and feces left in the metabolic cages were collected and kept at −80 °C until analysis.

All experiments were carried out by individuals with appropriate training and experience according to the requirements of the Federation of European Laboratory Animal Science Associations and the European Communities Council Directive (Directive 2010/63/EU). Experiments were approved by the regional authority (Regional Authority Darmstadt; V54 – 19 c 20/15 – FU/1056).

#### 2.3.3 Oral Application of ^13^C‐Labelled 2´FL to Germ‐Free Mice

Male C3H/HeN axenic (germ‐free) mice (6 weeks of age, 29–35 g body weight) supplied by Instituto Gulbenkian de Ciência (Oeiras, Portugal) were used in this study. Mice were kept in pairs and housed under standardized environmental conditions with free access to sterile water and autoclaved RM3A (P) diet (Special Diet Services, UK). Animals were kept in positive pressured individual ventilated cages (ISOcage P System from Tecniplast, Italy) located in a SPF (specific pathogen‐free) facility.

On the day of experiment, animals (total *n* = 12) received a single dose of 1 g ^13^C‐2´FL per kg body weight via gavage and were anesthetized after 5 or 12 h (*n* = 4 for each time point) using ketamine/xylacine (120/16 mg per kg body weight) whereas controls (*n* = 4) received water as the vehicle. After moving the animal from the isolator and into a vertical flow hood, the chest was opened and a blood sample was collected. Organ perfusion to remove all the blood from peripheral organs and sample collection was done as described above for wild‐type animals, but under sterile conditions. Brain, however, was separated into more sections such as cerebellum, hippocampus, frontal cortex, and striatum and remaining brain tissue. Additionally, some fecal pellets were collected upon animal delivery as well as at the end point of the study in order to verify the axenic status of the animals during the study. All the samples were kept at −80 °C until further analyses.

The national and institutional guidelines for the care and use of animals were followed, and the experimental procedures were reviewed and approved by the Ethics Committee for Animal Experiments and Animal Welfare Body of the Instituto Gulbenkian de Ciência (ethics registration code 0420/000/000/2011).

### 2.4 Analytical Methods

#### 2.4.1 Determination of ^13^C Enrichment by Elemental Analysis—Isotope Ratio Mass Spectrometry (EA‐IRMS)

Sample treatment and EA‐IRMS have been described in detail previously for various biological samples.^29^, ^32^, ^34^ Here, organs and tissues (brain regions, liver, heart, spleen, and kidney) were weighed and homogenized. All samples including plasma, urine, intestinal content, and feces were determined in triplicates. Aliquots of 0.5–20 mg, depending on their assumed water content, were weighed, transferred into tin capsules, and sealed. For liquid samples, a bed of acid‐washed Chromosorb W (IVA Analysentechnik, Meerbusch, Germany) was added as a sorbent. In the EA (Vario PyroCube from Elementar, Langenselbold, Germany), samples were combusted at 920 °C. Oxygen gas was injected in abundance and helium used as carrier gas. The oxidation reactor was filled with corundum, copper oxide, and elemental silver according to manufacturer's specifications (Elementar, Langenselbold, Germany). The combustion gas was then led into the reduction tube (650 °C, filled with corundum, elemental silver, and elemental copper) where NO*_x_* and other gases (but not CO_2_) were reduced and excess oxygen bound to the copper; the exiting gas stream was dried on phosphorus pentoxide. Generated CO_2_ from the samples was trapped and injected into the IRMS (Isoprime 100; Elementar UK, Stockport, UK) after appropriate dilution with helium. The amount of CO_2_ was quantified with a thermal conductivity detector and calculated as percentage of the original sample. For the sample peaks and the corresponding reference gas peaks the ion ratio (45/44) was quantified. The reference gases were calibrated against standard samples with known isotope‐amount ratio obtained from the International Atomic Energy Agency (IAEA), Vienna.

Elemental composition including blank correction for the tin capsules was calculated with the PyroCube software from Elementar (Langenselbold, Germany). All isotope ratio calculations were done using Elementar Software (IonVantage and Ionos; Elementar UK, Stockport UK) and results were expressed as δ^13^C_PDB_ enrichment as described earlier.^34^

### 2.5 Statistical Analysis

Statistical analyses were carried out using GraphPad Prism 6.0.7 (GraphPad Software Inc, La Jolla, USA) and results were expressed as box plots with medians and min to max whiskers or means with SD (standard deviation). Data were analyzed by ANOVA with multiple comparison test or Student's *t*‐test. Differences were considered significant at *p < 0.05, **p < 0.01, and ***p < 0.001.

## 3 Results

**Figure 1:**
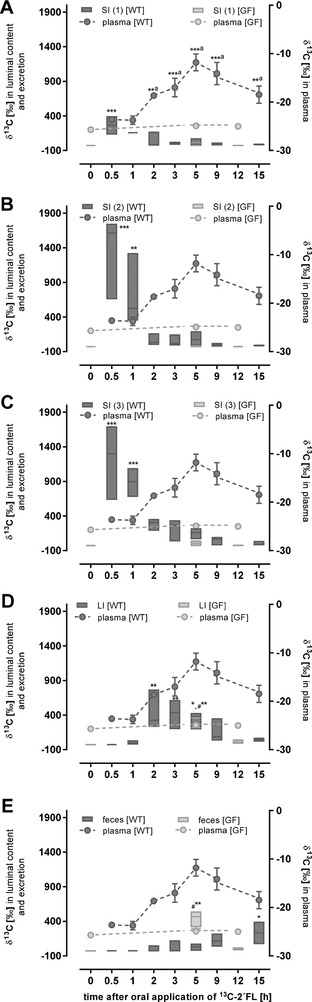
^13^C‐enrichment (δ^13^C in ^0^/_00_) in A–D) luminal contents of the different intestinal sections and E) feces (left axis) as well as in plasma (right axis). Wild‐type (WT; treated *n* = 5) and germ‐free mice (GF; treated *n* = 4) received 1 g per kg body weight ^13^C‐labelled 2´FL by oral gavage. After indicated time points, mice were sacrificed and luminal contents, feces, and plasma were collected and ^13^C‐enrichment was measured by EA‐IRMS. Values are depicted as box plots with median and min–max whiskers; data for plasma (dashed line) are shown as mean ± SD. Differences to initial time points were significant at **p < 0.01 and ***p < 0.001 for plasma (A) and differences to corresponding controls were *p < 0.05, **p < 0.01, and ***p < 0.001 for intestinal parts of the SI (SI (1), SI (2), and SI (3)) and the LI for WT mice and #**p < 0.01 for LI and feces for GF.

**Figure 2:**
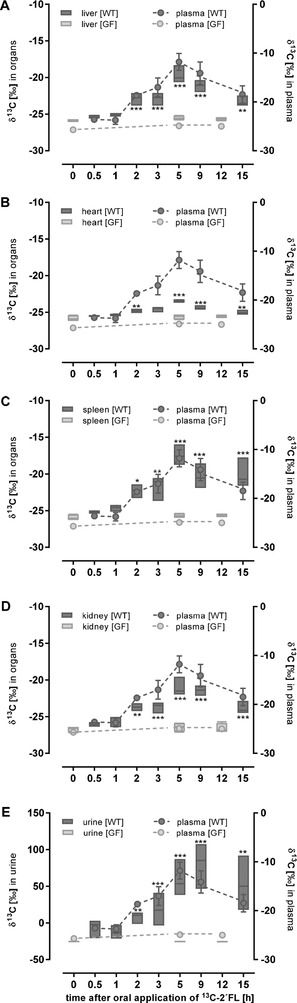
^13^C‐enrichment (δ ^13^C in ^0^/_00_) in organs and plasma (left axis) and urine (right axis). Wild‐type (WT; treated *n* = 5) and germ‐free mice (GF; treated *n* = 4) received 1 g per kg body weight ^13^C‐labelled 2´FL by oral gavage. After the indicated time points, mice were sacrificed and organs, plasma, and urine were collected and ^13^C‐enrichment was measured by EA‐IRMS. Data are depicted as box plots with median and min–max whiskers; data for plasma are shown as mean ± SD (differences to corresponding controls were significant at *p < 0.05, **p < 0.01, and ***p < 0.001).

**Figure 3:**
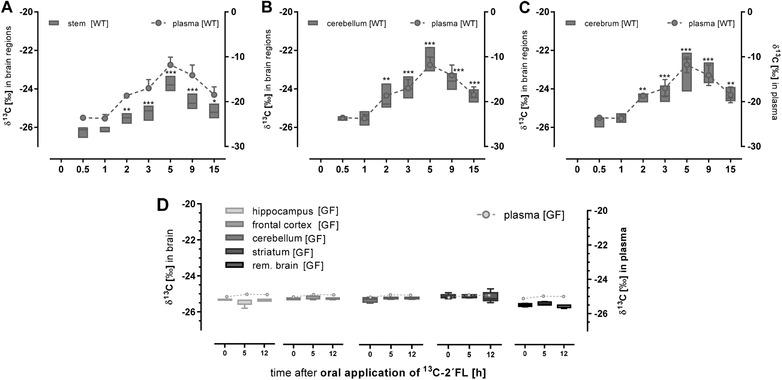
^13^C‐enrichment (δ ^13^C in ^0^/_00_) in brain sections (left axis) and plasma (right axis). Wild‐type (WT; treated *n* = 5) and germ‐free mice (GF; treated *n* = 4) received 1 g per kg body weight ^13^C‐labelled 2´FL by oral gavage. After the time points indicated, mice were sacrificed and the brain of A–C) WT mice was separated in stem, cerebellum, and cerebrum and D) of GF micein hippocampus, striatum, frontal cortex, cerebellum, and remaining brain. ^13^C‐enrichment was measured by EA‐IRMS. Data are depicted as box plots with median and min–max whiskers; data for plasma are shown as mean ± SD (differences to corresponding controls were significant at **p < 0.01 and ***p < 0.001).

To measure the metabolic fate of 2´FL in vivo, ^13^C‐labelled 2´FL at 1g per kg body weight was given to wild‐type and germ‐free mice by oral gavage, whereas the corresponding control groups received the vehicle only. ^13^C‐enrichment is shown in **Figure** 1 for luminal contents of the small intestine, separated into three equal parts, the large intestine, feces, and plasma. The intestinal transit of ^13^C‐2´FL occurred rapidly, with the maximal ^13^C‐enrichment in the first two SI segments 1 h after oral administration of the bolus (Figure 1A–C). Already after 2 h, ^13^C‐enrichment was observed in the LI and peaked after 3 h (Figure 1D). In feces, ^13^C‐enrichment was low in the first 2–3 h and then increased steadily over time (Figure 1E). Comparing the ^13^C‐enrichment of the three parts of the small intestine and the colon with δ^13^C_PDB_‐values in plasma (Figure 1) and organs (**Figure** 2) including the brain (**Figure** 3A–C), it is apparent that most of the ^13^C‐labelled 2´FL was fermented by the microbiota present in the lower part of the intestine. This assumption is supported by the ^13^C‐accumulation in plasma, which reached the highest level after 5 h concomitantly with the increase in ^13^C‐enrichment of the LI.

Comparing the results of the wild‐type mice having an intact microbiota with those of the germ‐free mice two main observations are notable. First, 5 h after the oral dose, increased δ^13^C_PDB_–values were only detected in the LI and feces indicating a fast transit of ^13^C‐2´FL (Figure 1D,E). Second, no significant ^13^C‐enrichment was seen neither in plasma nor in organs of germ‐free mice (Figure 2). However, in liver, heart, spleen, and kidney of wild‐type mice, ^13^C‐enrichment increased 2 h post‐dosing and peak accumulation was observed at 5 h (Figure 2A–D). ^13^C‐levels in urine 9 h post‐consumption were lower than those found in feces, indicating that feces was the primary route of elimination (Figure 2E). In germ‐free animals, no ^13^C‐enrichment could be observed, neither in organs nor in urine (Figure 2A–E).

After oral ^13^C‐2´FL application,^13^C‐enrichment in all brain sections of wild‐type mice increased significantly compared to controls, but remained at a low level (Figure 3A–C). The time course was in parallel to the increase in plasma and other organs starting to rise after 2 h and reaching its maximum after 5 h. Again, in the germ‐free mouse model, no ^13^C‐enrichment could be detected in any of the brain sections (Figure 3D).

**Figure 4:**
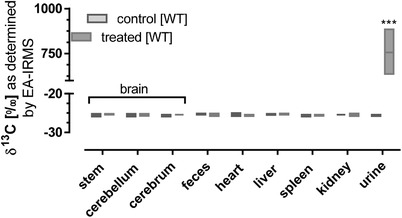
^13^C‐enrichment (δ ^13^C in ^0^/_00_) in brain sections, organs, and urine. WT mice received intravenously 0.2 g per kg body weight ^13^C‐labelled 2´FL. 12 h after the last of three partial dosages, mice were sacrificed and organs and urine were collected as described in the section materials and methods (treated *n* = 5, controls *n* = 3). Data are depicted as box plots with median and min–max whiskers (differences to corresponding controls were significant at ***p < 0.001).

In contrast to the observation in wild‐type mice receiving ^13^C‐2´FL by oral gavage, intravenous administration did not reveal a ^13^C‐enrichment in any body compartment, and was only found in urine (**Figure** 4).

## 4 Discussion

HMO may have a variety of beneficial effects for the infant. Currently, an influence on neurodevelopmental outcomes including intelligence and behavioral performance in animal studies has been reported.^28^ The proposed association is based on observations that breast‐fed infants had better cognitive performances, memory functions, and intelligence quotients (IQ) than formula‐fed infants.^22^, ^43^, ^44^, ^45^, ^46^ Furthermore, fucose and fucosylated glycans (fucose‐α1–2‐galactose epitopes) are implicated in the mechanisms that underlie neuronal development, and glycosylation influences various neuronal processes.^26^, ^27^, ^47^ Thus, it is of particular interest to investigate whether 2´FL, the most predominant HMO in milk of secretor positive mothers,^5^, ^7^ or its metabolites reach the circulation and finally enters the brain to serve as a substrate for neurites.

In animals, limited information about the absorption of 2´FL and its distribution in various organs is available. Jantscher‐Krenn et al. detected minor amounts of 2´FL in urine of neonatal rats after receiving an HMO‐mix with 2´FL being the most abundant component.^48^ In rats that were given high doses of 2´FL (5 g per kg body weight), Vazquez et al. observed a fast 2´FL enrichment in serum after 30 min with a concentration of approximately 30 µg mL^−1^.^49^ At this time point, neither fucose nor lactose could be detected. Only after 6 h, they found a significant increase of lactose, but not fucose. The authors suggested that 2´FL was fermented by the intestinal microbiota to lactose which was also detected in blood. In contrast to the low serum concentrations, they reported high quantities of 2´FL as well as of lactose and fucose in urine.^49^

By using ^13^C‐labelled 2´FL in wild‐type and germ‐free mice to address metabolic aspects, we showed that 2´FL itself was not incorporated into the brain. This conclusion is based on four main observations: First, after oral application of ^13^C‐labelled 2´FL to germ‐free mice, neither a systemic ^13^C‐enrichment nor a ^13^C‐enrichment in any organ including the brain was observed. Second, oral application of ^13^C‐labelled 2´FL to wild‐type mice was associated with a significant ^13^C‐enrichment in systemic compartments but an organ with a preferential ^13^C‐enrichment was not detected. Third, the ^13^C‐enrichment after 2 h in plasma and organs correlated with the fast ^13^C‐enrichment in the lower segments of the gut, especially in the lower small and large intestine. Fourth, no ^13^C‐accumulation in organs could be observed after ^13^C‐labelled 2´FL was applied intravenously. Here, ^13^C‐2´FL was excreted via the animals´ urine. Altogether, we speculate that systemic ^13^C‐enrichment in plasma and organs including the brain in wild‐type mice with an intact microbiota is a consequence of intestinal microbial activity toward 2´FL. In their previously used rat model, Vazquez et al. have shown that 2´FL can be cleaved, most likely by the gut microbiota, to lactose and fucose.^28^ Hence, in our study with ^13^C‐2´FL with the label only at C‐atom 1 of 2´FL, the released fucose still being ^13^C‐labelled on C‐atom 1 may actually enter the circulation and finally reach the brain. However, ^13^C‐fucose may also be metabolized to ^13^C_1_‐dihydroxy‐acetone‐phosphate and lactataldehyde. The latter is known to be a precursor of 1,2 propane‐diol which may then be metabolized to propionate,^50^ a bacterial metabolite which may activate gut–brain neural signaling.^51^ Another possibility could be that the gut–brain communication through the vagus nerve can be influenced by 2´FL as has been suggested,^28^, ^49^ but this effect was not addressed in our experiments.

Recent research indicates that signaling molecules generated by the microbiota seem to mediate some of the observed effects on brain development and behavior via the gut–brain axis.^52^, ^53^, ^54^, ^55^, ^56^ A different metabolic profile generated by the microbiota of breast‐fed infants compared to formula‐fed infants has been identified by Chow et al. using GC/MS and LC/MS/MS.^57^ Since we applied a single ^13^C‐2´FL dose in short‐term experiments up to 15 h, the activity of individual microorganisms already present in the gut rather than a change in the microbial pattern appears to be more important with regard to the production of metabolites. In vitro, some *Bifidobacteria* species such as *Bifidobacterium longum subsp. infantis* and *B. bifidum* have been shown to grow well in 2´FL containing culture media, whereas others, for example, *Bifidobacterium animalis*, do not.^35^ Interestingly, growth responses are not only species but even strain dependent. For example, some *Bifidobacteria* strains (e.g., *B. bifidum* SC555) were not able to induce key genes for the HMO metabolism such as fucosidases (α‐fucosidase), which would be responsible for the cleavage of 2´FL to fucose and lactose.^35^, ^41^, ^58^ However, 2´FL may also serve as substrate for *Bacteroides*, which are able to express fucosidases and degrade 2´FL to fucose and lactose.^59^ Interestingly, 2´FL was able to induce the expression of alternative operons distinct from HMO cluster I.^58^, ^60^ This could be an important point since the lack of fucosylated oligosaccharides in mouse milk may lead to a lower level of fucosidase‐expressing bacteria in their gut. Although there is an intensive debate about the comparability of the mouse microbiome with that of the human,^61^, ^62^ some mouse‐associated species such as *Bacteroides* were able to metabolize HMO. For example, *Bacteroides thetaiotaomicron* has been previously shown to consume HMO via mucin O‐glycan degradation pathways.^63^ Furthermore, species of the genus *Barnesiella*, namely *Barnesiella intestinihominis*, were able to utilize FL.^64^, ^65^ Interestingly, 2´FL application to mice increases the levels of *Barnesiella* indicating that in mice 2´FL metabolization is a possible route to generate metabolites which could cross the intestinal barrier and accumulate in the circulation. In this context, the consumption of 2´FL by microbial species from the genera *Barnesiella, Bifidobacterium*, and *Bacteroides* may lead to the production of smaller molecules such as lactose, fucose or organic acids such as butyrate, acetate, or lactate.^57^, ^66^, ^67^ With regard to our experiments, we speculate that ^13^C‐enrichment may reflect the presence of fucose itself or a fucose‐derived metabolite since the fucose moiety of 2´FL carried the ^13^C‐labeling at the C_1_‐atom and 2´FL itself could not pass the blood–brain barrier as has been shown by i.v. experiments. Whether the presence of microbial metabolites in organs such as the brain are necessary for 2´FL to exert biological functions or whether an indirect stimulation via gut–brain signaling is effective remains unclear and requires further studies.

## 5 Concluding Remarks

The present study demonstrates for the first time that after the application of ^13^C‐labelled 2´FL, ^13^C‐fucose, or a fucose metabolite carrying the ^13^C‐label, most likely generated by the intestinal microbiota of mice, are responsible for the ^13^C‐enrichment in the systemic circulation and organs, as opposed to native 2´FL. ^13^C‐enrichment was detectable in liver, heart, spleen, kidney, and brain of wild‐type mice, indicating that there is no specific uptake of 2´FL into any organ.

These results suggest that 2´FL itself does not reach the brain. Thus, the direct incorporation of intact 2´FL into the brain does not appear to be required to affect learning processes observed in previous animal studies.

## Conflict of Interest

S.K., C.K., C.B., M.R., G.P.E., and S.R. declare no conflict of interest. R.B. and E.V. are currently working for Abbott Nutrition, which is a company that manufactures infant formula. Other than that, there are no known conflicts of interest associated with this publication.
